# Supplementary material for: Biofilm Formation among Stenotrophomonas maltophilia Isolates Has Clinical Relevance: The ANSELM Prospective Multicenter Study
Source: Microorganisms. 2020 Dec 27;9(1):49. doi: 10.3390/microorganisms9010049 (PMC7823565; doi:10.3390/microorganisms9010049)
Supplement: Supplementary file 1 [file microorganisms-09-00049-s001.pdf]

Article

Biofilm formation among *Stenotrophomonas maltophilia* isolates has clinical relevance: the ANSELM prospective multicenter study

Arianna Pompilio, et al.

Supplementary materials

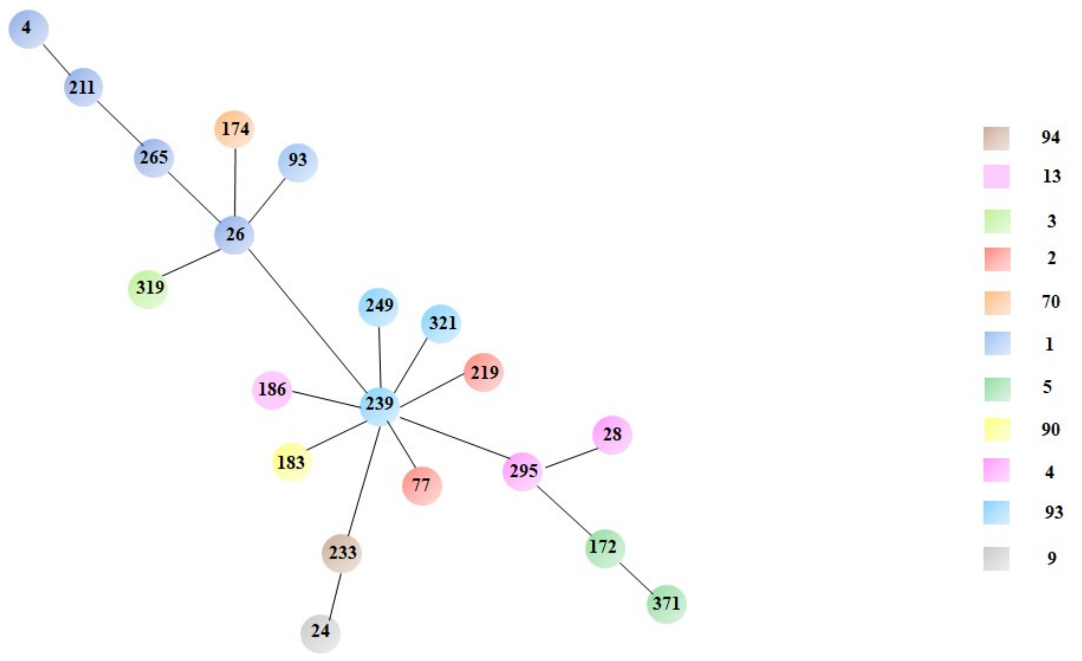

Figure S1. Phylogenetic tree of *S. maltophilia* strains based on *atpD* MLST profiles. Minimum spanning tree obtained using PHLOViZ Online software. The different categories of *atpD* are indicated with different colors.

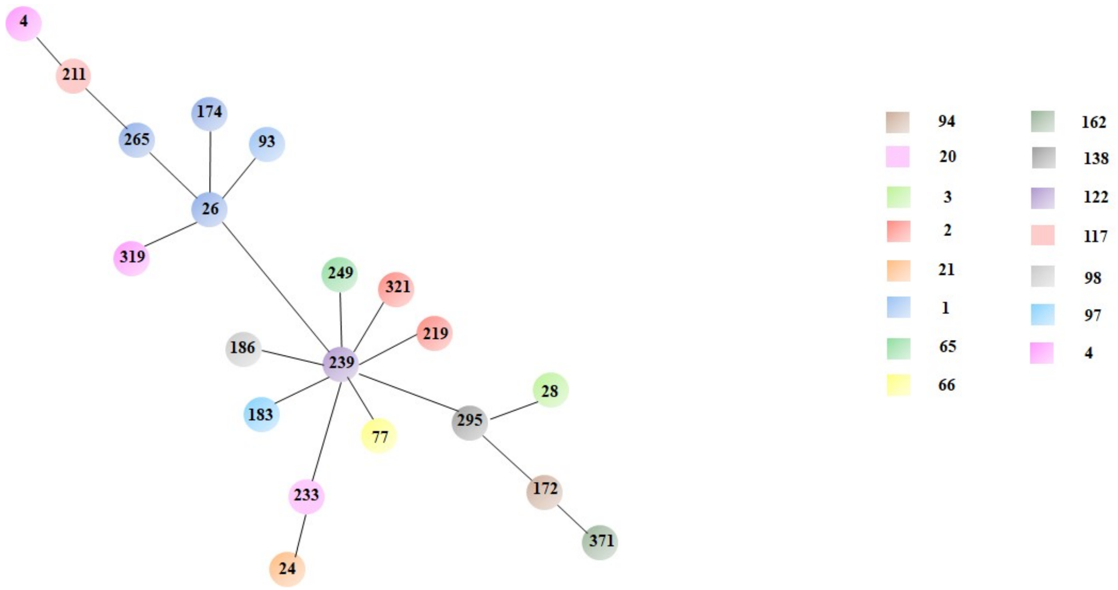

Figure S2. Phylogenetic tree of *S. maltophilia* strains based on *gapA* MLST profiles. Minimum spanning tree obtained using PHLOViZ Online software. The different categories of *gapA* are indicated with different colors.

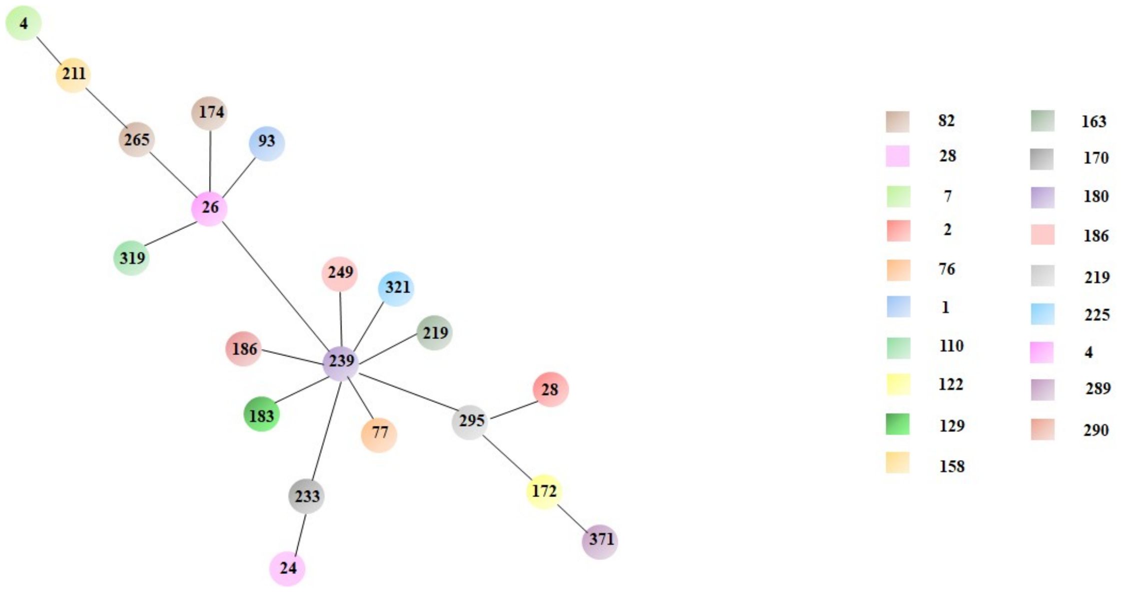

Figure S3. Phylogenetic tree of *S. maltophilia* strains based on *guaA* MLST profiles. Minimum spanning tree obtained using PHLOViZ Online software. The different categories of *guaA* are indicated with different colors.

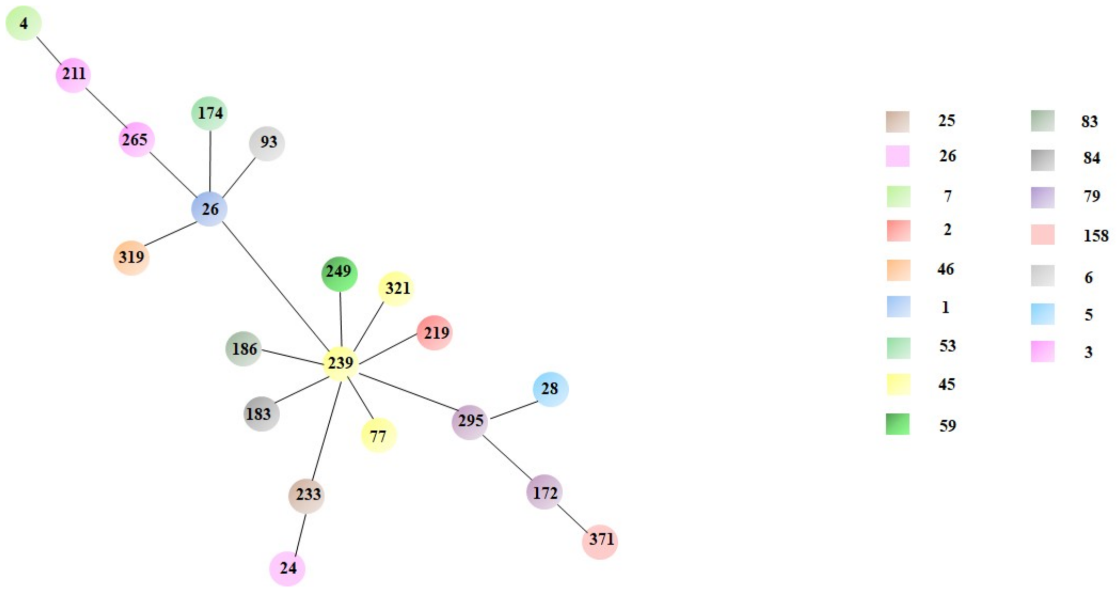

Figure S4. Phylogenetic tree of *S. maltophilia* strains based on *mutM* MLST profiles. Minimum spanning tree obtained using PHLOViZ Online software. The different categories of *mutM* are indicated with different colors.

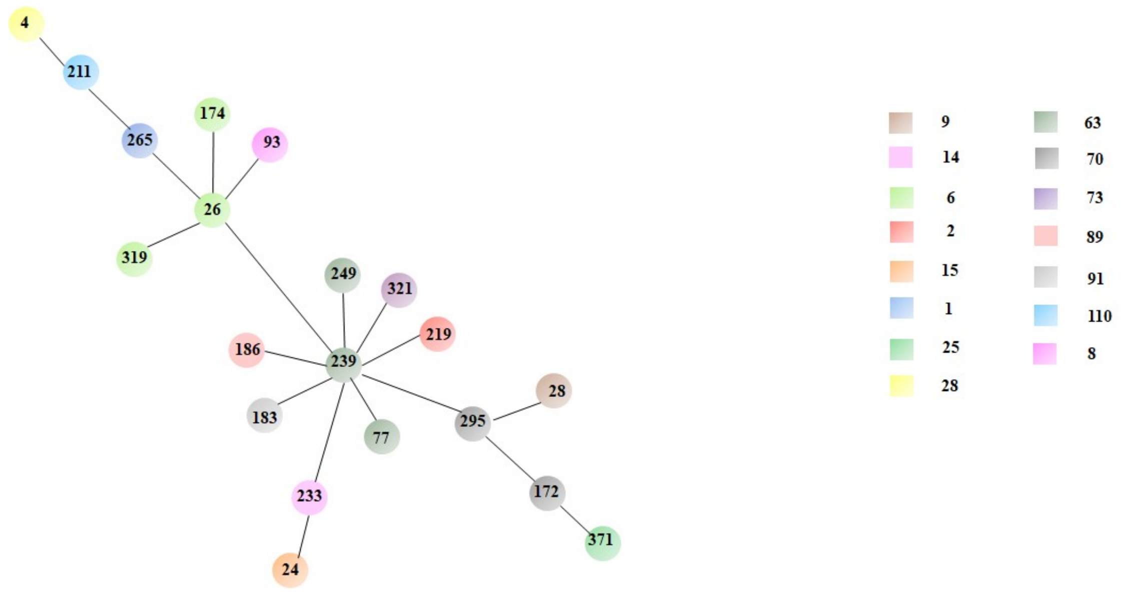

Figure S5. Phylogenetic tree of *S. maltophilia* strains based on *nuoD* MLST profiles. Minimum spanning tree obtained using PHLOViZ Online software. The different categories of *nuoD* are indicated with different colors.

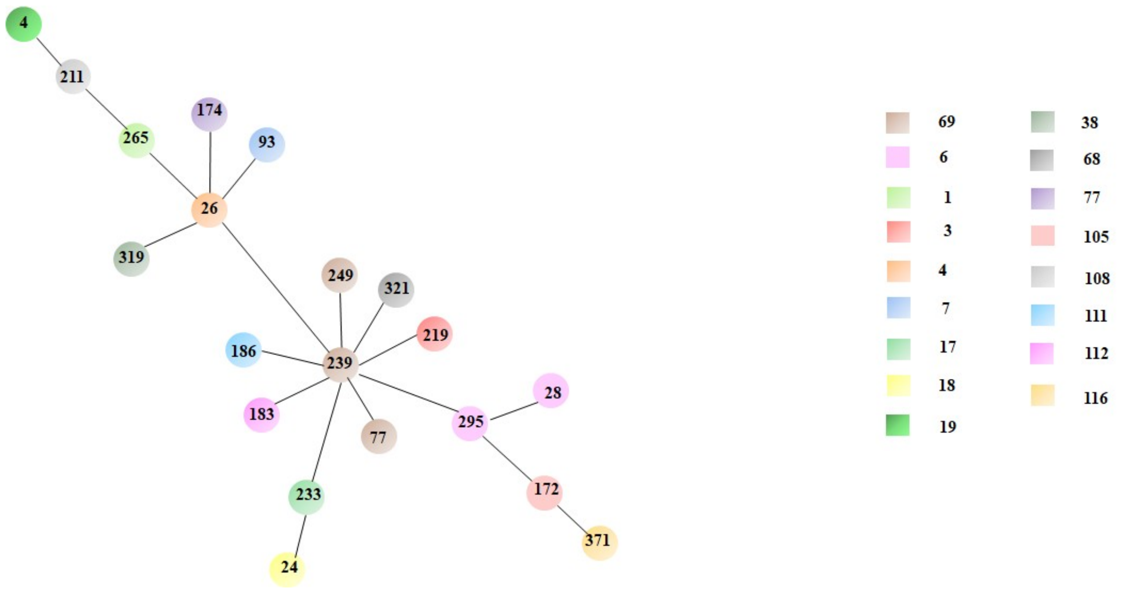

Figure S6. Phylogenetic tree of *S. maltophilia* strains based on *ppsA* MLST profiles. Minimum spanning tree obtained using PHLOViZ Online software. The different categories of *ppsA* are indicated with different colors.

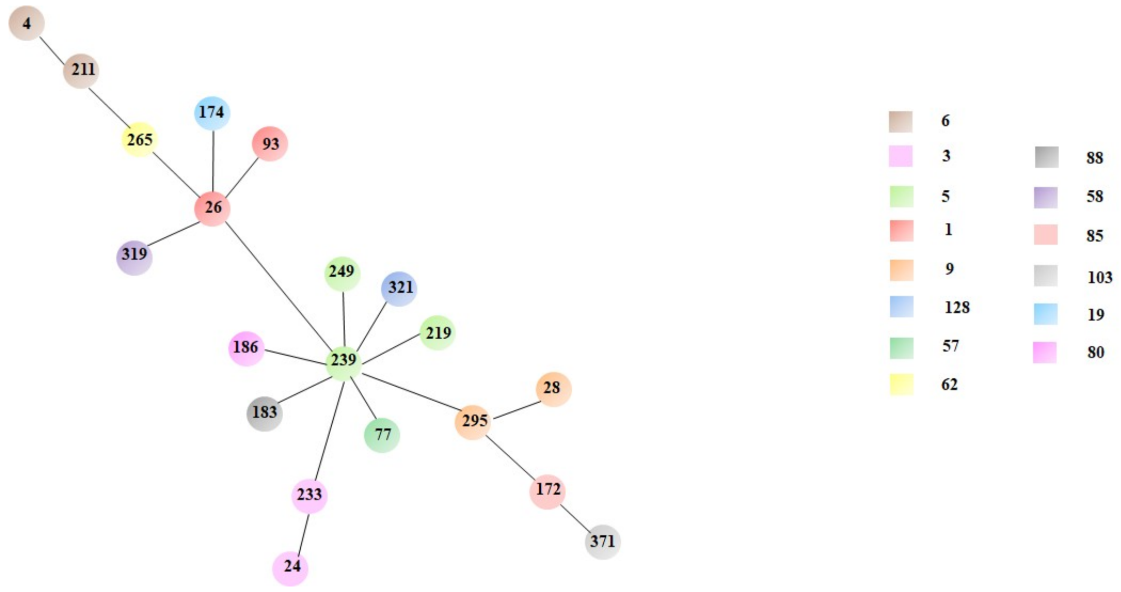

Figure S7. Phylogenetic tree of *S. maltophilia* strains based on *recA* MLST profiles. Minimum spanning tree obtained using PHLOViZ Online software. The different categories of *recA* are indicated with different colors.

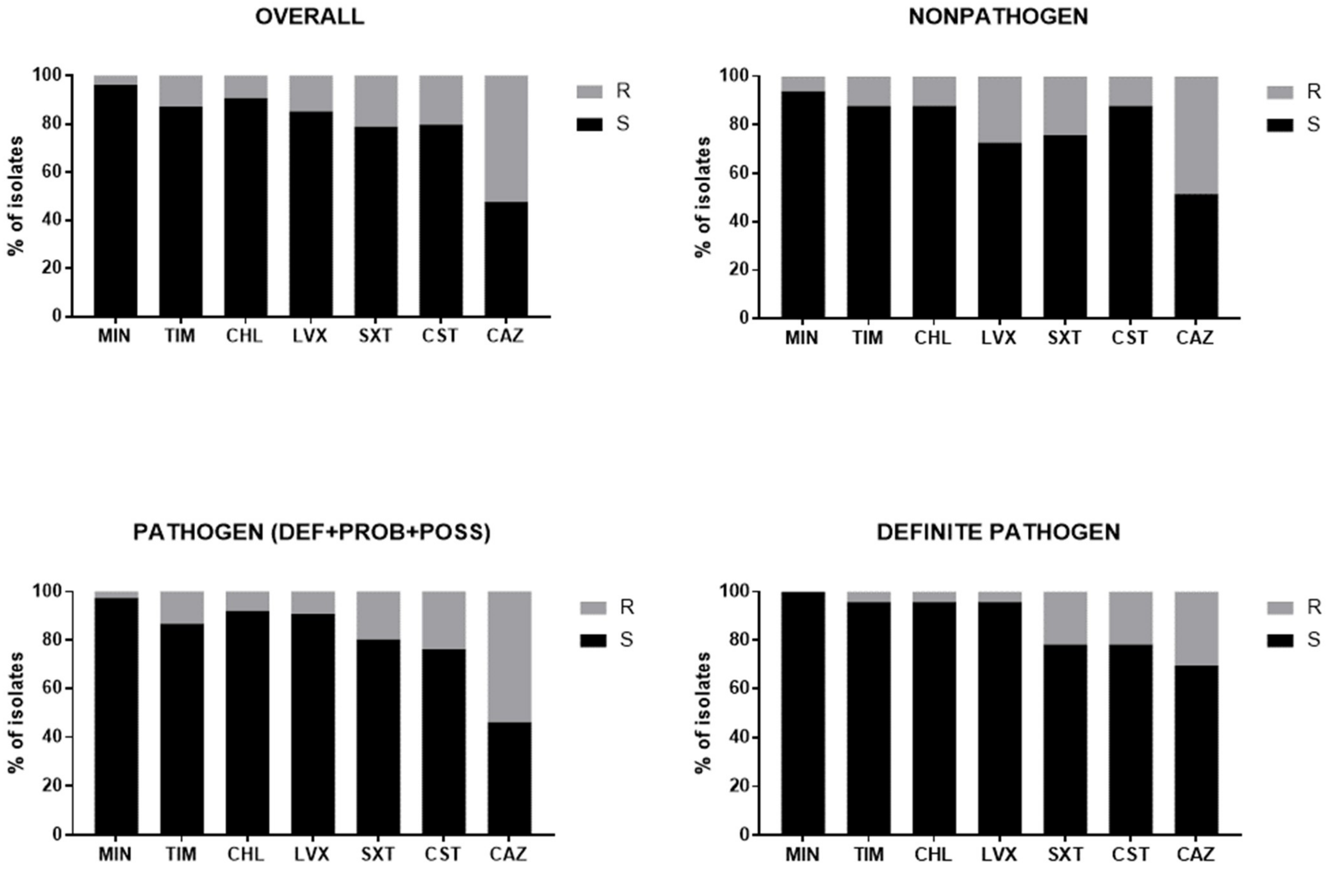

Figure S8. In vitro susceptibility to antibiotics by *S. maltophilia*. The activity of seven antibiotics (MIN, minocycline; TIM, ticarcillin-clavulanic acid; CHL, chloramphenicol; LVX, levofloxacin; SXT, trimethoprim-sulfamethoxazole; CST, colistin; CAZ, ceftazidime) was evaluated by the disk diffusion method according to CLSI guidelines [25]. Results were stratified according to the strains' pathogenic role (definite, probable, possible, and non-pathogen), defined in accordance with the CDC guidelines [22]. R, resistant and intermediate strains; S, susceptible strains.

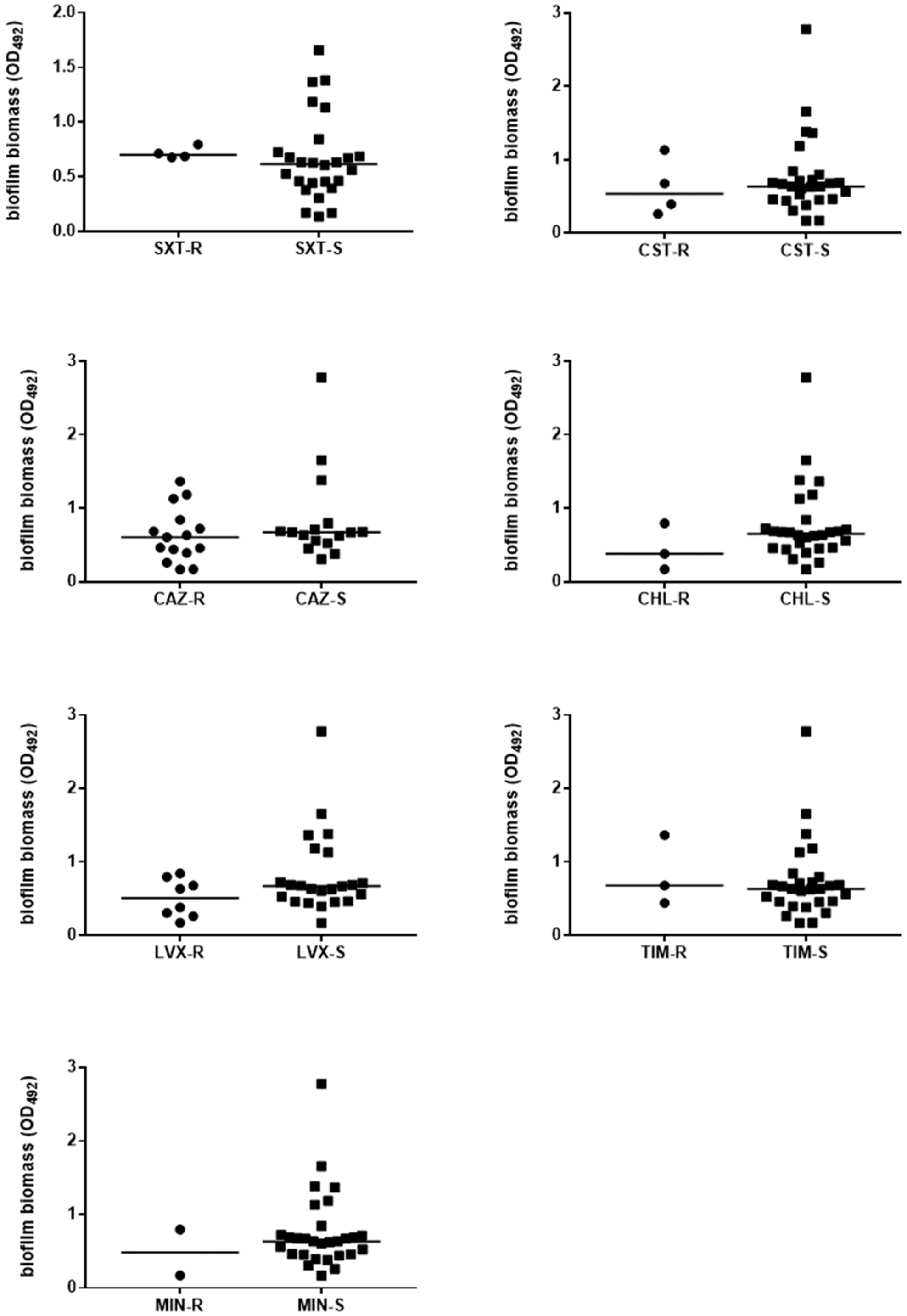

Figure S9. Biofilm formation by *S. maltophilia* non-pathogenic strains: stratification on the susceptibility phenotype. Non-pathogenic strains – defined according to the CDC guidelines [22] – were tested for biofilm formation in a 96-well polystyrene microtiter by crystal violet assay after a 24 h-incubation at 37°C. Results were stratified on the susceptibility or resistance to each antibiotic tested (SXT, trimethoprim-sulfamethoxazole; CST, colistin; CAZ, ceftazidime; CHL, chloramphenicol; LVX, levofloxacin; TIM, ticarcillin-clavulanic acid; MIN, minocycline) evaluated by the disk diffusion technique. Each dot shows the mean OD_{492} value, with the horizontal line indicating the median value of each distribution. No statistically significant differences were found using a Kruskal-Wallis test followed by a Dunn's multiple comparison post-test.

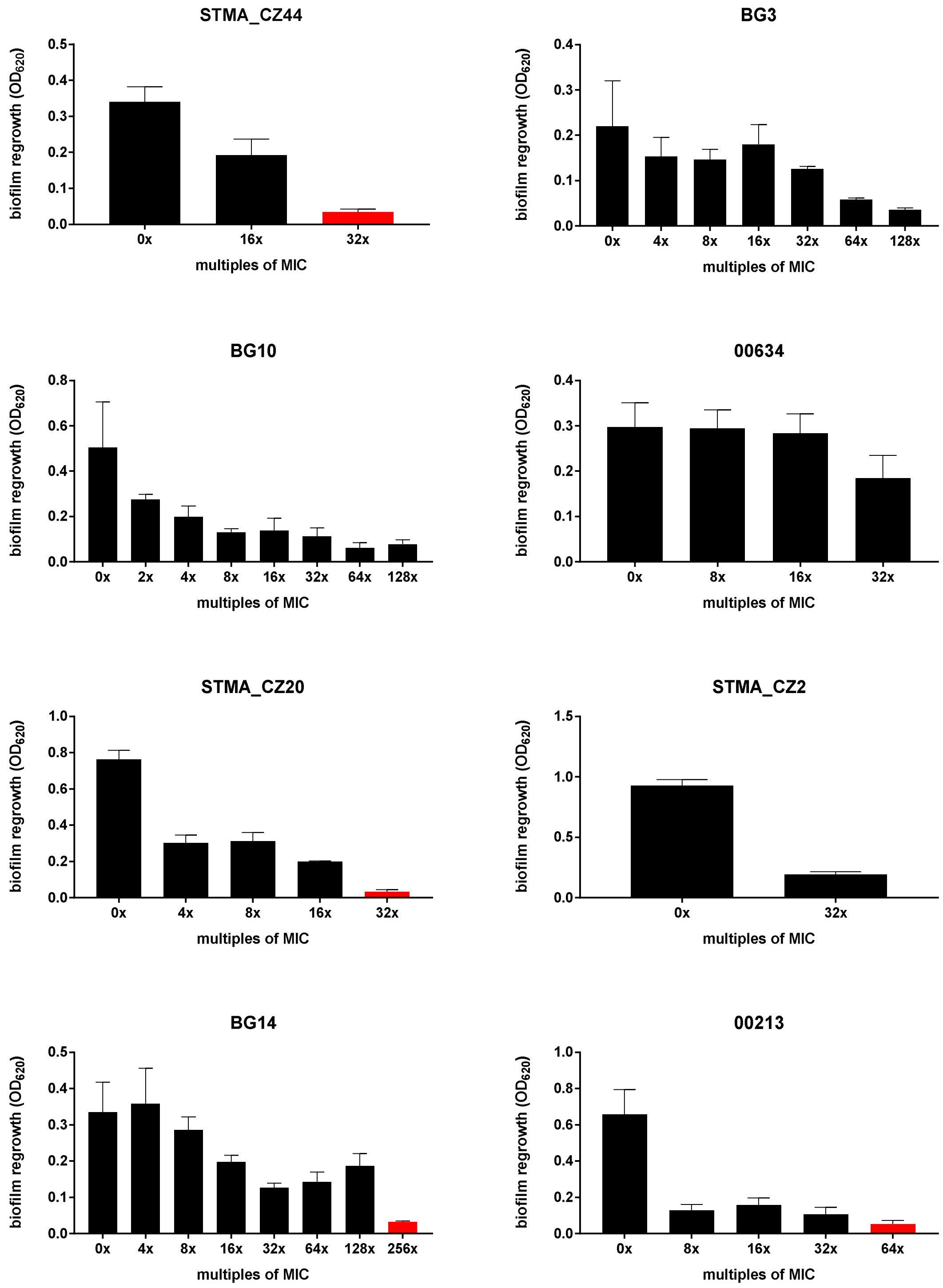

Figure S10 … *continued…*

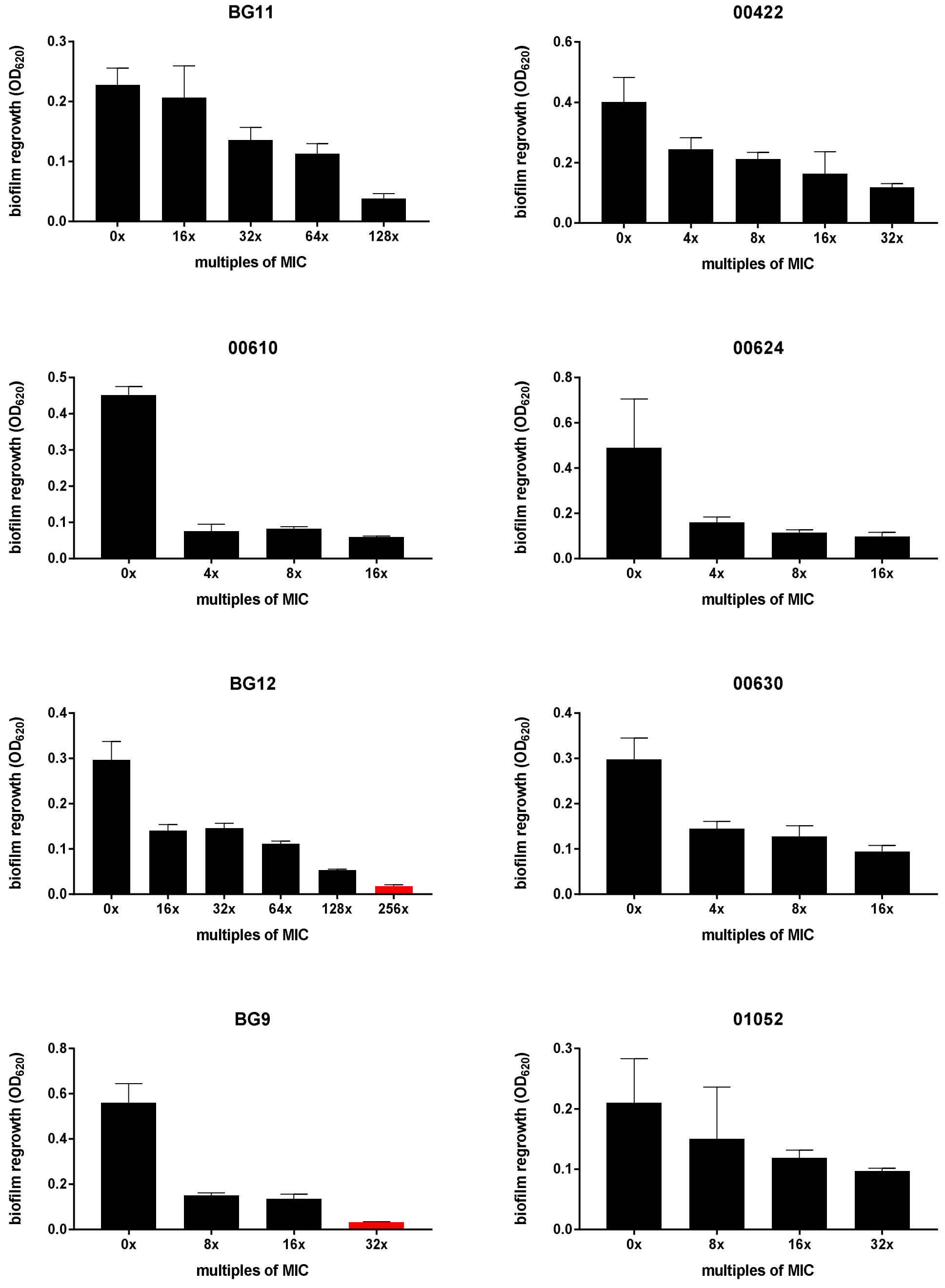

Figure S10. In vitro activity of cotrimoxazole against preformed biofilms by *S. maltophilia*: Minimum Biofilm Inhibitory Concentration (MBIC). Mature 24 h-old biofilms were exposed for 20 h to cotrimoxazole at several multiples of the MIC. Biofilm samples were washed and then fresh broth was added to verify the biofilm viability. After a 6 h-incubation at 37°C, the OD_{620} of broth supernatant was read. The control sample (0x) was not exposed to the antibiotic. The MBIC values are highlighted in red and defined as the lowest antibiotic concentration allowing a regrowth of ≤ 10% compared to the control well readings. Results are expressed as the mean + SD.

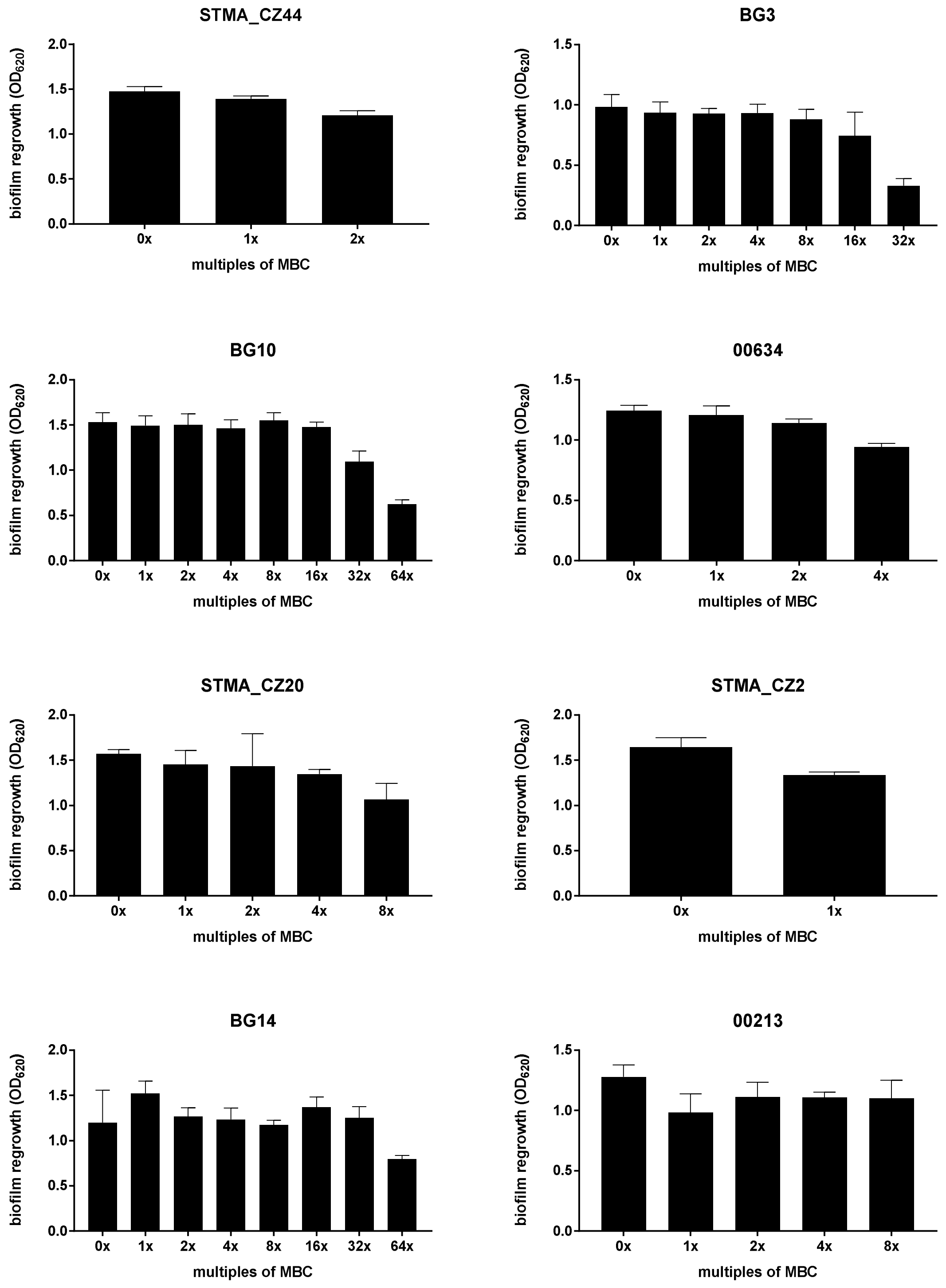

Figure S11 … *continued…*

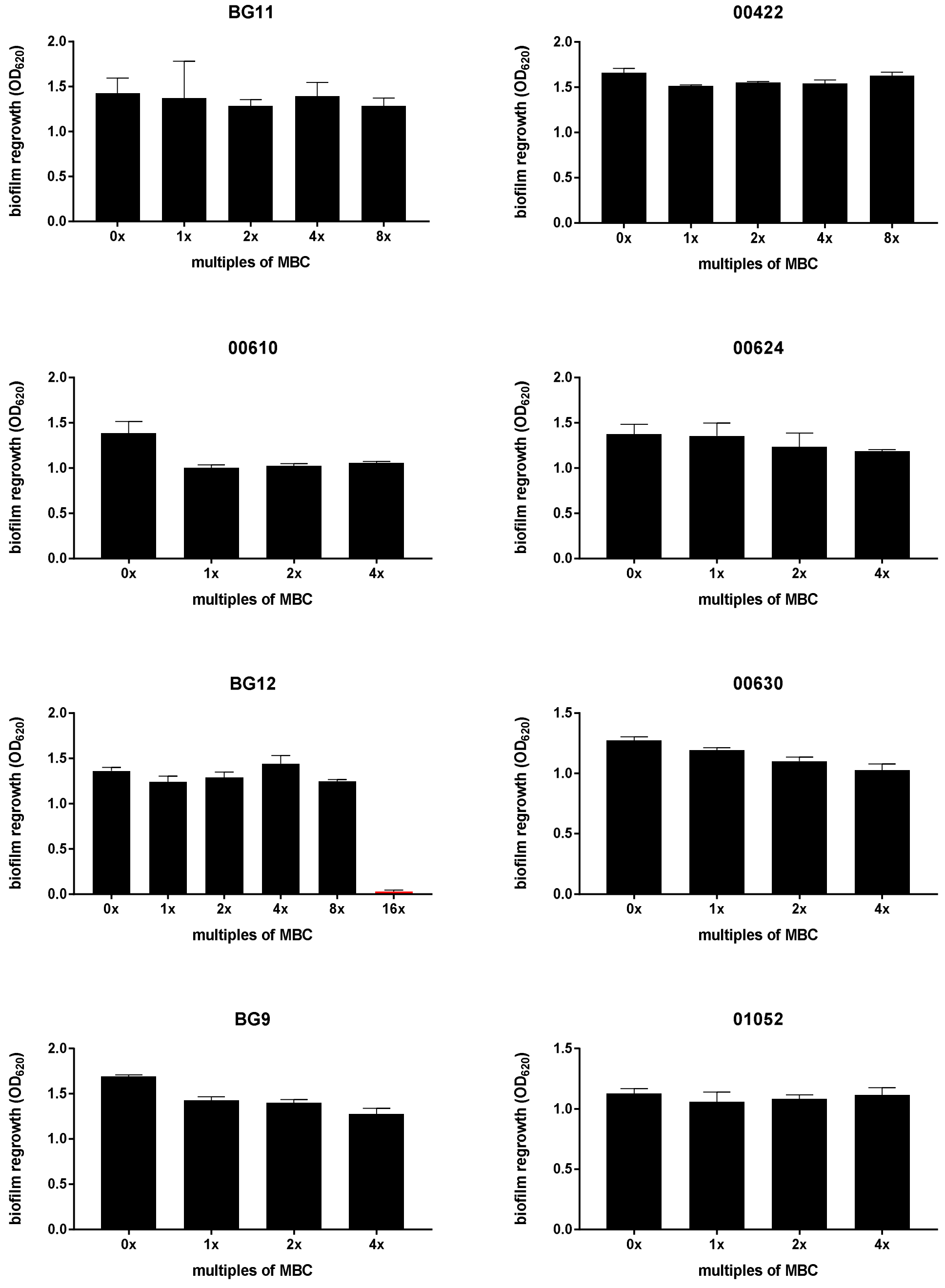

Figure S11. In vitro activity of cotrimoxazole against preformed biofilms by *S. maltophilia*: Minimum Biofilm Eradication Concentration (MBEC). Mature 24 h-old biofilms were exposed for further 20 h to cotrimoxazole at several multiples of the MBC. Biofilm samples were washed, and then fresh broth was added to verify the biofilm viability. After a 24 h-incubation at 37°C, OD_{620} of broth supernatant was read. The control sample (0x) was not exposed to the antibiotic. The MBEC values are highlighted in red and defined as the lowest antibiotic concentration allowing biofilm eradication compared to the control well readings. Results are expressed as the mean + SD.

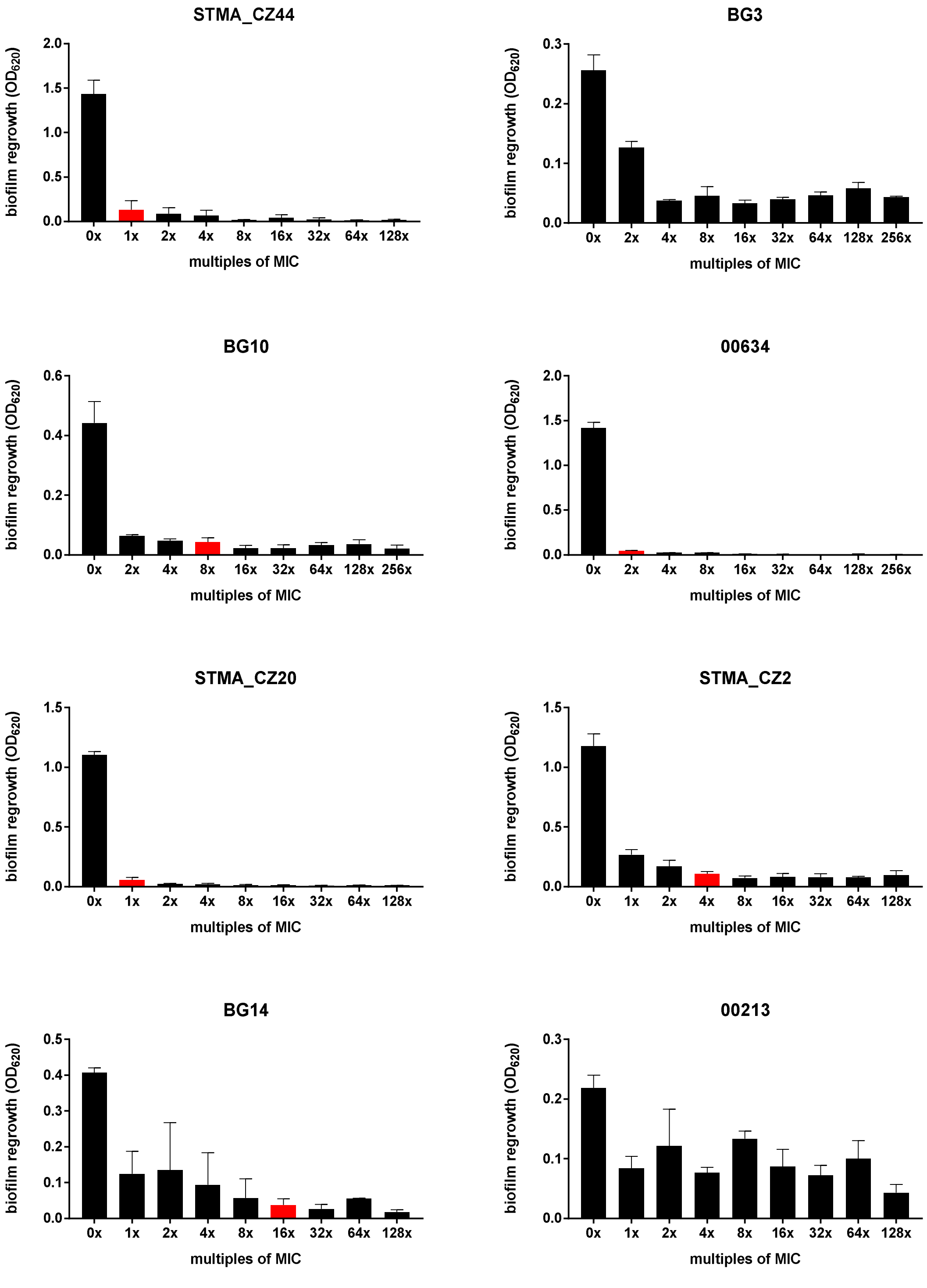

Figure S12 … *continued…*

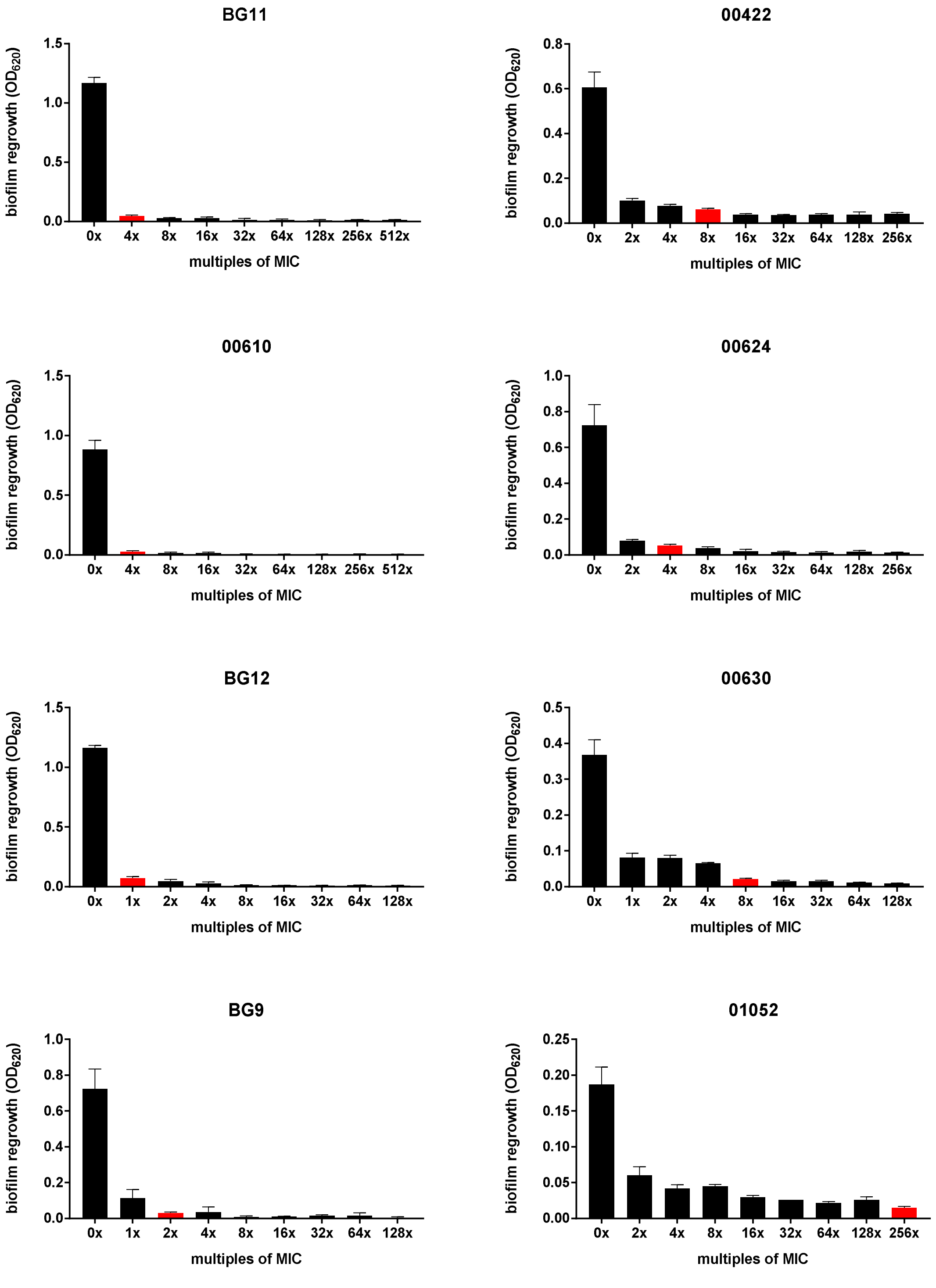

Figure S12. In vitro activity of levofloxacin against preformed biofilms by *S. maltophilia*: Minimum Biofilm Inhibitory Concentration (MBIC). Mature 24h-old biofilms were exposed for further 20 h to levofloxacin at several multiples of the MIC. Biofilm samples were washed and then fresh broth was added to verify the biofilm viability. After a 6 h-incubation at 37°C, the OD_{620} of broth supernatant was read. The control sample (0x) was not exposed to the antibiotic. The MBIC values are highlighted in red and defined as the lowest antibiotic concentration allowing a regrowth of ≤ 10% compared to the control well readings. Results are expressed as the mean + SD.

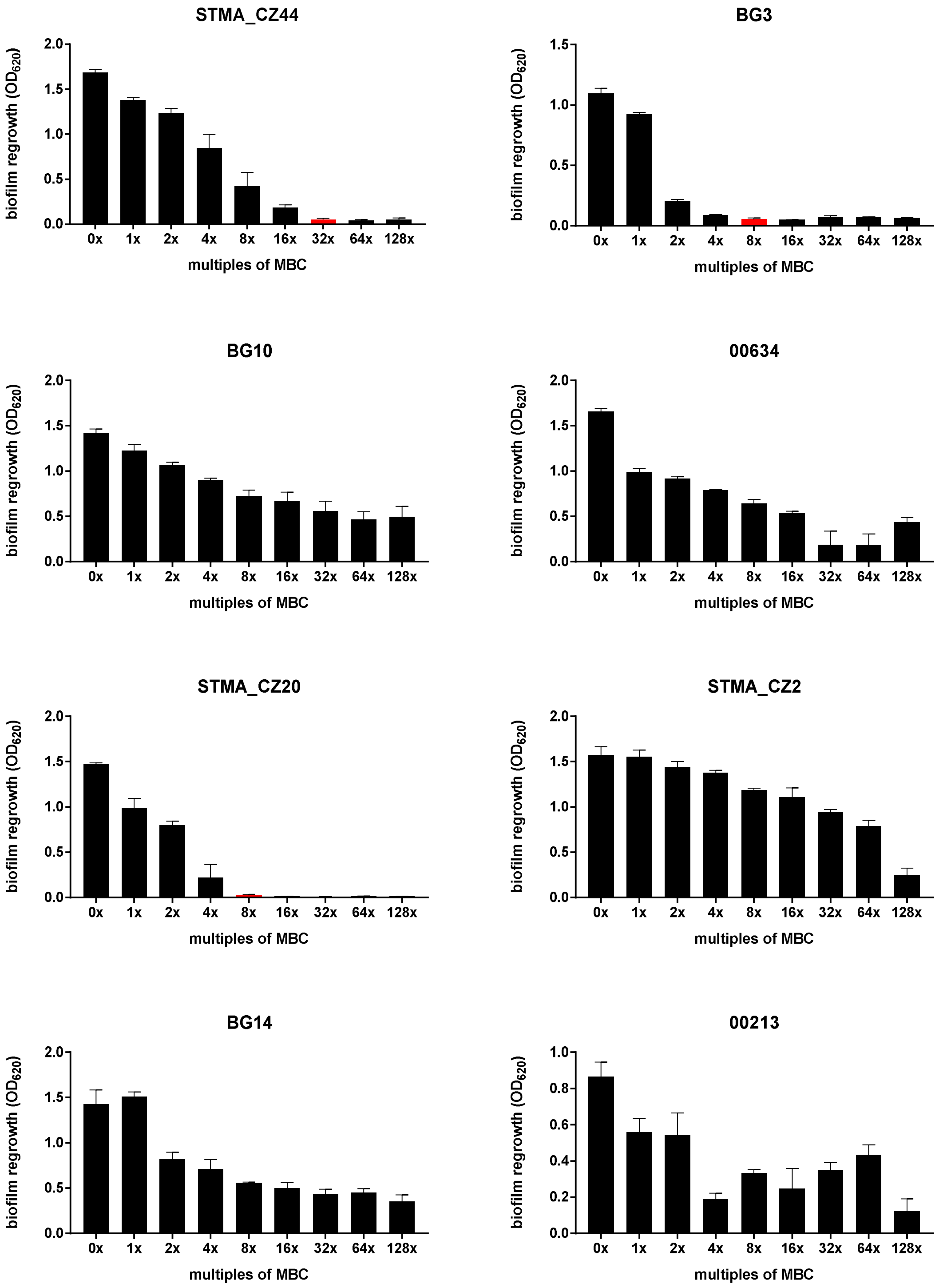

Figure S13 … *continued…*

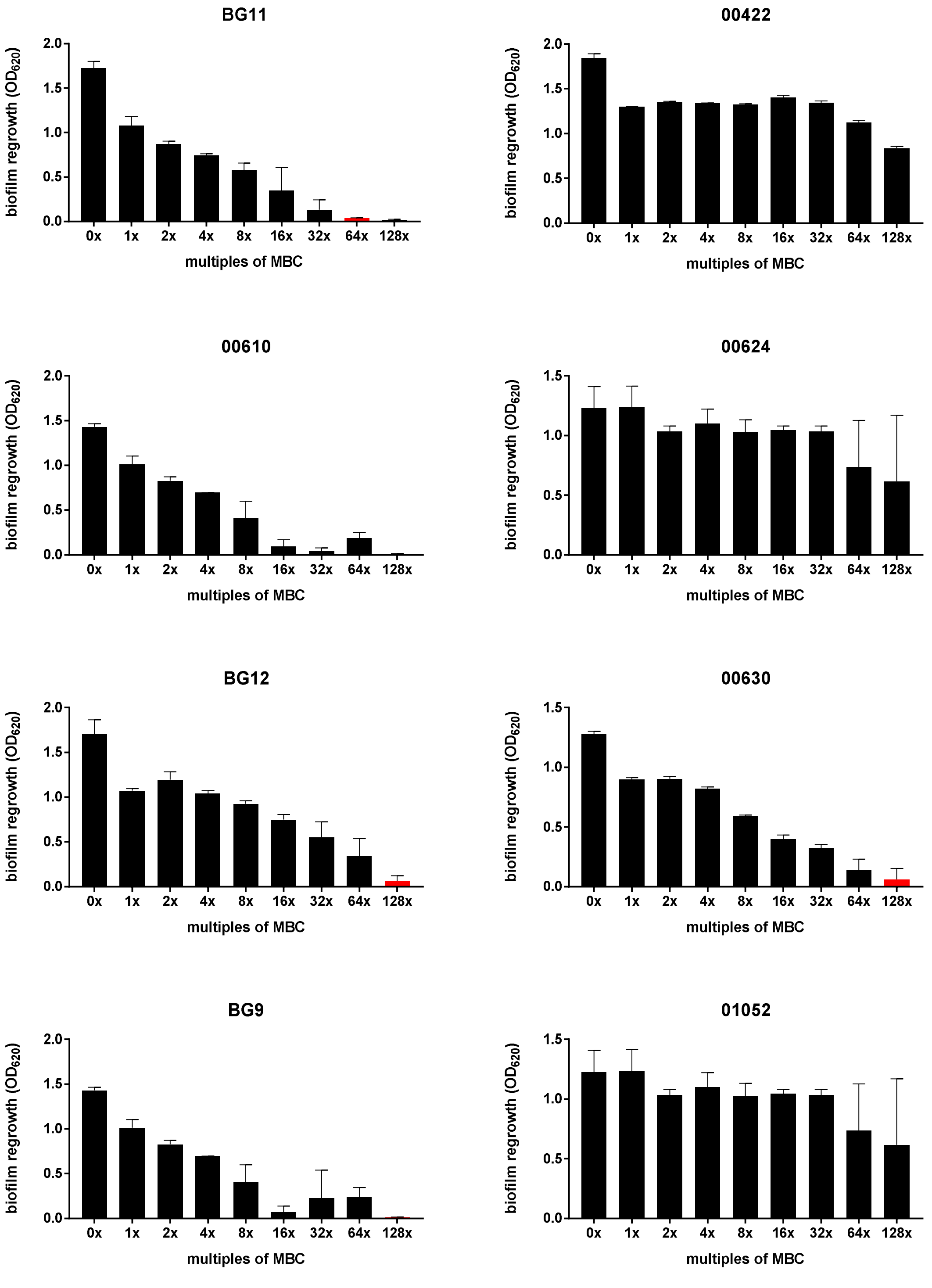

Figure S13. In vitro activity of levofloxacin against preformed biofilms by *S. maltophilia*: Minimum Biofilm Eradication Concentration (MBEC). Mature 24 h-old biofilms were exposed for further 20 h to levofloxacin at several multiples of the MBC. Biofilm samples were washed, and then fresh broth was added to verify the biofilm viability. After a 24 h-incubation at 37°C, the OD_{620} of broth supernatant was read. Control sample (0x) was not exposed to the antibiotic. The MBEC values are highlighted in red and defined as the lowest antibiotic concentration allowing biofilm eradication compared to the control well readings. Results are expressed as the mean + SD.
